# A Novel Design and Development of a Strip-Fed Circularly Polarized Rectangular Dielectric Resonator Antenna for 5G NR Sub-6 GHz Band Applications

**DOI:** 10.3390/s22155531

**Published:** 2022-07-25

**Authors:** Usman Illahi, Javed Iqbal, Muhammad Irfan, Mohamad Ismail Sulaiman, Muhammad Abbas Khan, Abdul Rauf, Inam Bari, Mujeeb Abdullah, Fazal Muhammad, Grzegorz Nowakowski, Adam Glowacz

**Affiliations:** 1 Electrical Engineering Department, FET, Gomal University, Dera Ismail Khan 29050, KP, Pakistan; javediqbal.iet@gu.edu.pk; 2 School of Electrical and Electronic Engineering, Engineering Campus, Universiti Sains Malaysia, Nibong Tebal 14300, Penang, Malaysia; 3 Electrical Engineering Department, College of Engineering, Najran University, Najran 61441, Saudi Arabia; miditta@nu.edu.sa; 4 Advanced Telecommunication Technology, Communication Technology Section, British Malaysian Institute, Universiti Kuala Lumpur, Gombak 53100, Selangor, Malaysia; mismail@unikl.edu.my; 5 Department of Electrical Engineering, Balochistan University of Information Technology, Engineering and Management Sciences, Quetta 87300, Pakistan; muhammad.abbas@buitms.edu.pk; 6 Department of Electrical Engineering, National University of Sciences and Technology, H-12, Islamabad 44000, Pakistan; a.rauf@nust.edu.pk; 7 Systems Engineering Department, Military Technological College, Muscat 111, Oman; inam.bari@mtc.edu.om; 8 School of Aeronautical and Electrical Engineering, College of Aeronautical Engineering, National University of Science and Information Technology, Risalpur 24080, Pakistan; mabdullah@cae.nust.edu.pk; 9 Electrical Engineering Department, University of Engineering and Technology, Mardan 23200, Pakistan; 10 Faculty of Electrical and Computer Engineering, Cracow University of Technology, Warszawska 24 Str., 31-155 Kraków, Poland; gnowakowski@pk.edu.pl; 11 Department of Automatic Control and Robotics, Faculty of Electrical Engineering, Automatics, Computer 22 Science and Biomedical Engineering, AGH University of Science and Technology, A. Mickiewicza 30, 23-059 Kraków, Poland; adglow@agh.edu.pl

**Keywords:** dielectric resonator antennas, strip-fed antennas, axial ratio, 5G NR band, Sub-6 GHz

## Abstract

In this article, a rectangular dielectric resonator antenna (RDRA) with circularly polarized (CP) response is presented for 5G NR (New Radio) Sub-6 GHz band applications. A uniquely shaped conformal metal feeding strip is proposed to excite the RDRA in higher-order mode for high gain utilization. By using the proposed feeding mechanism, the degenerate mode pair of the first higher-order, i.e., ${\mathrm{TE}}_{\delta 13}^{x}$ at 4.13 GHz and ${\mathrm{TE}}_{1\delta 3}^{y},\;$ at 4.52 GHz is excited to achieve a circularly polarized response. A circular polarization over a bandwidth of ~10%, in conjunction with a wide impedance matching over a bandwidth of ~17%, were attained by the antenna. The CP antenna proposed offers a useful gain of ~6.2 dBic. The achieved CP bandwidth of the RDRA is good enough to cover the targeted 5G NR bands around 4.4–4.8 GHz, such as n79. The proposed antenna configuration is modelled and optimized using computer simulation technology (CST). A prototype was built to confirm (validate) the performance estimated through simulation. A good agreement was observed between simulated and measured results.

## 1. Introduction

The 5G New Radio (NR) is a newly developed air interface to fulfill the requirements of modern communications. 5G wireless technology is becoming popular because of its significant features, such as high data rate, low latency response time, and high bandwidth [1]. The 5G NR is a multi-band spectrum and n79 (4.4–4.8 GHz) is one of the major focuses for future 5G communications in different parts of the world, such as China, the EU, and Japan [2]. In literature, different 5G antennas have been reported so far [3,4]. In the last few decades, the dielectric resonator antenna (DRA) has been acquiring a lot of attention from antenna researchers. This is due to their significant benefits over conventional microstrip antennas. DRAs offer high bandwidth, negligible metal losses, small size, and fixable excitation techniques which make them a potential candidate for high-frequency modern networks, such as 5G [5,6,7,8,9,10,11,12].

DRAs can be designed in different shapes for desired applications but complicated shapes add complexity and design configurations are expensive. So basic dielectric resonator (DR) shapes, such as rectangular, cylindrical, and hemispherical are much more popular because of ease in fabrication and low cost. Among these basic shapes, rectangular is much preferred because of design flexibility. In the rectangular shape, the height to width and depth to width aspect ratios have a direct impact on resonance frequency and Q-factor of the radiation. Moreover, using the dielectric waveguide model (DWM), the DR profile and impedance bandwidth characteristics of the rectangular DRA can be predicted [13].

In this era of modern communication, reliability of the communication system is highly important. That is why circularly polarized communication networks are very much preferred as compared to linearly polarized communication networks [3]. A lot of circularly polarized DRAs are reported in the literature using dual feeding techniques, but with limitations, such as complex feeding networks and larger sizes, which make them useless for modern communication [14,15,16]. The generation of circular polarization in DRA using a single feeding network is quite challenging. Some authors performed cutting and drilling of the dielectric resonator, some used multi-layered DRs, having metallic walls surrounding the RDRA, and more than one DR has been reported as well. All such arrangements are complex, heavy, and costly [17,18,19,20].

Different DRAs using a single feeding mechanism have been reported in the literature [18,19,20,21,22]. In [18], a multi-layered rectangular DRA, excited by a cross-slot coupled with a microstrip feeding mechanism providing 9.5% of CP response, has been reported. As demonstrated in [19], a feeding technique comprised of cross-slot and microstrip was used to generate a 46.9% CP wave by a rectangular DRA surrounded by four metallic walls. As presented by M. Elahi et al., in [20], a 3 dB axial ratio bandwidth of 5.5% was attained by using two annular vias (holes) in a rectangular DR excited by a rectangular slot coupled microstrip line feed. As reported by A. Gupta et al., in [21], the CP bandwidth of 2.29% is achieved using rectangular DR excited by a triangular ring-shaped aperture with a parasitic patch. A cross-slot coupled with a microstrip line to attain CP bandwidth of 2.2% using rectangular-shaped DR was reported in [22]. In all discussed literature a sufficient bandwidth of impedance matching was achieved to cover the attained AR bandwidth. However, it is clarified that the generation of circular polarization using a single feeding mechanism with simple design geometry is quite challenging and a solution to this problem is reported in this article. 

In this research paper, a new feeding mechanism, using a uniquely shaped conformal metal strip, is introduced to excite the rectangular DRA for CP response. The proposed feeding technique excites the degenerate higher-order mode pair to generate the circularly polarized wave. A prototype was built to study the experimental results. Antenna parameters, such as impedance matching, axial ratio, beamwidth, and gain, were studied theoretically and experimentally. A reasonable similarity was observed in all antenna parameters. The proposed antenna could be used for 5G Sub-6 GHz communications around 4.4–4.8 GHz, such as n79 [2]. In Section 2, antenna design geometry is explained. In Section 3, antenna design, optimization, and CP generation mechanism are discussed in detail. In Section 4 comparison between experiment and theory is demonstrated and explained. Section 5 features the conclusion to the article.

## 2. Antenna Geometry

**Figure 1:**
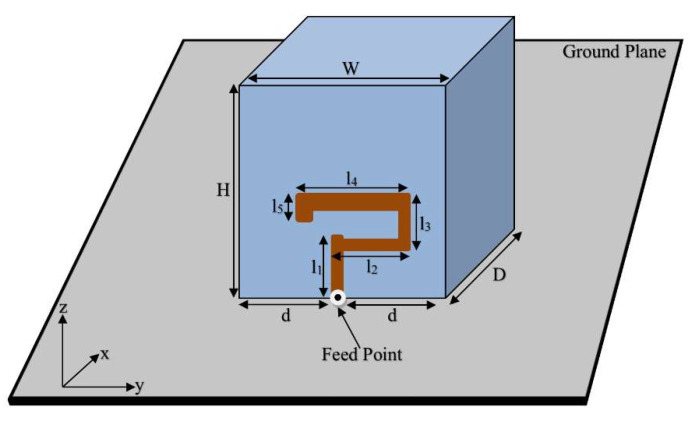
The geometry of the proposed CP 5G RDRA.

Figure 1 presents the configuration of the proposed geometry of the circularly polarized antenna excited by a uniquely shaped conformal strip placed with a feed point at the central position. The design was modeled in CST^®^ Microwave Studio using its time domain-based finite integration technique [23]. Hexahedral meshing was used to design a rectangular DRA with DR permittivity, $\varepsilon_{r}=10$ [11]. In settings, the cell per wavelength was set at a value of 40 and the cells per max model box edge at 20 for design mashing. The fraction of maximum cell near to model was also set at a value of 20 and total unknowns of 559,980 were obtained. 

An iterative design procedure was followed to determine the optimum dimensions of the feeding metallic strips that were needed to excite the degenerate ${\mathrm{TE}}_{\delta 13}^{x}$ and ${\mathrm{TE}}_{1\delta 3}^{y}$ necessary for CP wave generation [24]. The DR profile dimensions of H = 26.1 mm, W = 25.4 mm, and D = 14.3 mm were used. The optimization of the lengths and the widths of the strip were performed by running different simulations using many parameter sweeps. The results of the design procedure are summarized in Table 1, which shows several selected dimensions of *l_1_*, *l_2_*, *l_3_*, *l_4_*, and *l_5_* that could generate circular polarization in conjunction with sufficient impedance matching bandwidth.

## 3. Circularly Polarized 5G Antenna Design and Optimization

In this section, the design and development of CP 5G NR Sub-6 GHz RDRA are explained in detail. Moreover, the antenna optimization, along with results, is discussed. The lengths and widths of the feeding strip were very critical in this design configuration which is herein demonstrated and discussed. It is a well-known fact that a circularly polarized wave can be generated if the feeding network is capable of exciting the orthogonal degenerate modes, since such excitation generates two far-field components that are equal in magnitude with the quadrature phase shift necessary for CP generation [25]. In this section an initially linearly polarized rectangular DRA was designed using a single conformal strip. The next feeding mechanism was modified step by step to design a circularly polarized DRA without adding any complicated changes. Five different design geometries for the development of CP 5G Sub-6 GHz RDRA are presented and discussed in detail. 

### 3.1. Geometry 1

**Figure 2:**
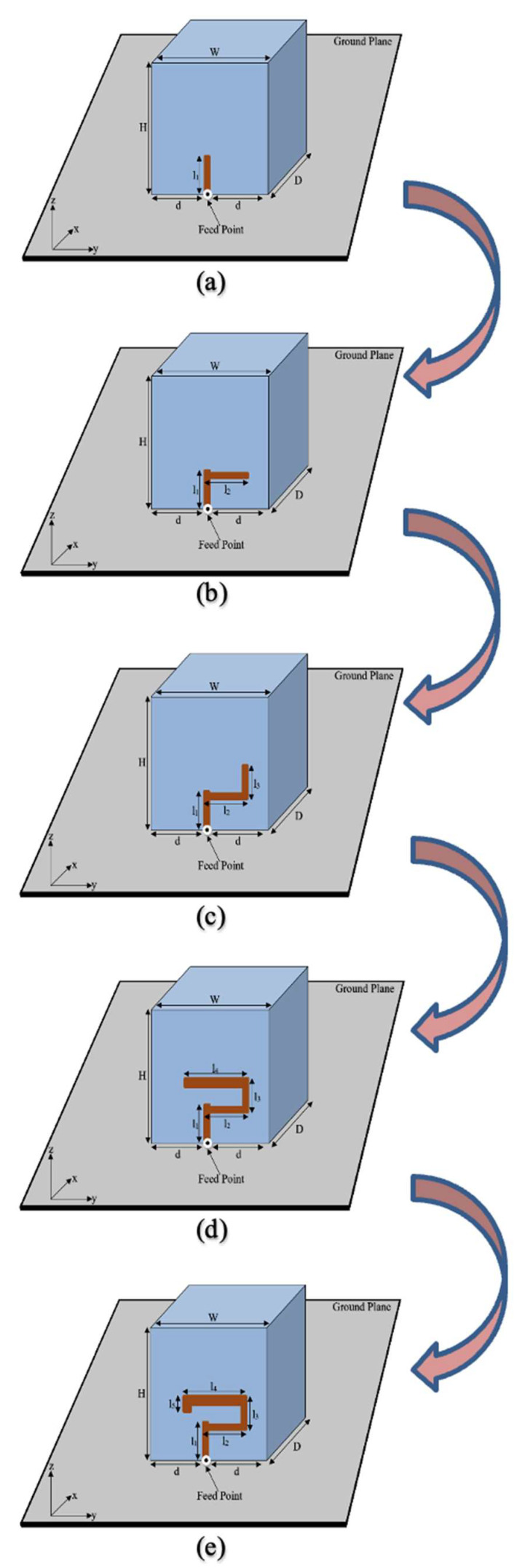
Step by step development of the CP 5G RDRA. (**a**) Geometry 1; (**b**) Geometry 2; (**c**) Geometry 3; (**d**) Geometry 4; (**e**) Geometry of the Proposed CP 5G RDRA.

**Figure 3:**
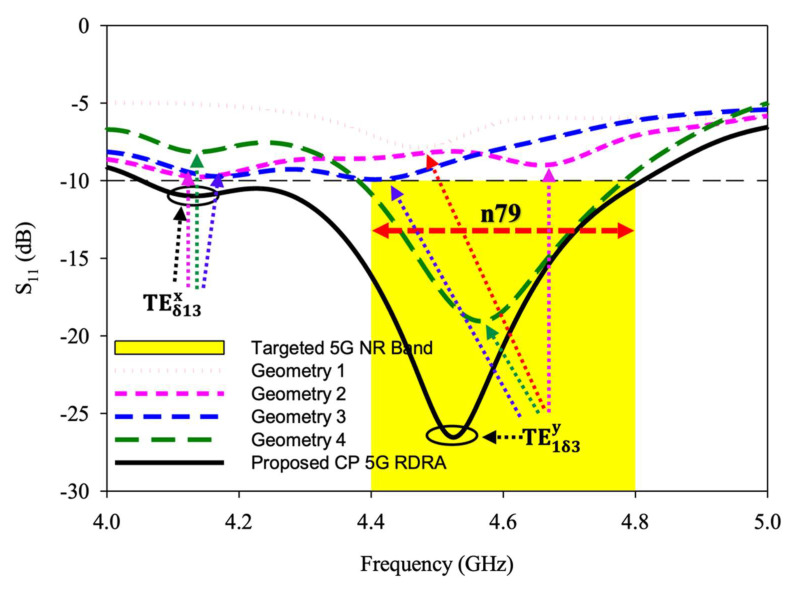
Return losses comparison of different geometries toward the development of the CP 5G RDRA.

**Figure 4:**
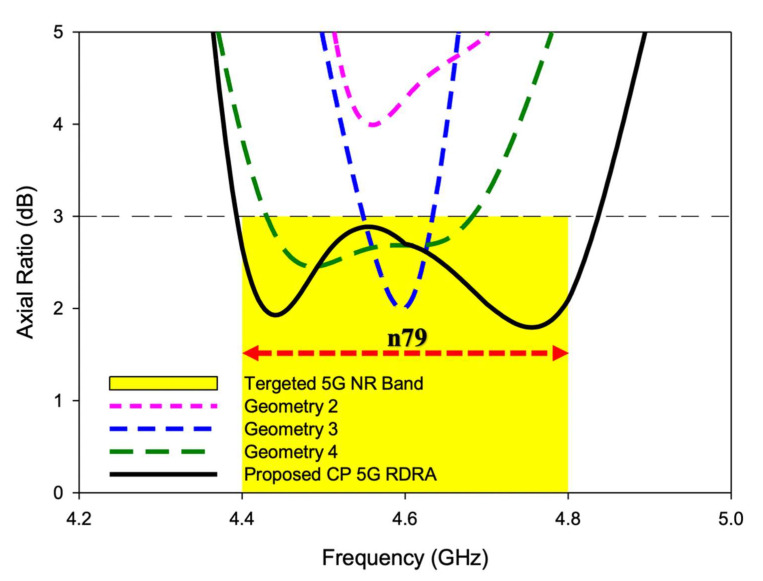
Axial ratios comparison of different geometries toward the development of the CP 5G RDRA.

In Figure 2a geometry 1 of RDRA is depicted. As shown, the antenna was designed by placing a single conformal strip at the middle of the surface of the DRA. The antenna was linearly polarized because such excitation does not excite the degenerate mode. Only ${\mathrm{TE}}_{1\delta 3}^{y}\;$ was energized, at around 4.47 GHz, to generate a linearly polarized wave, as shown in Figure 3. The result of the axial ratio of geometry 1 is not shown in Figure 4.

### 3.2. Geometry 2

The geometry 2 of RDRA is shown in Figure 2b. The feeding strip was modified by adding one more length. Using this configuration, the RDRA showed behavior towards CP response. The degenerate mode was excited but still, S_11_ bandwidth below −10 dB was nil, as shown in Figure 3. As presented in Figure 3, the degenerate mode pair of the first higher-order, i.e., ${\mathrm{TE}}_{\delta 13}^{x}$ and ${\mathrm{TE}}_{1\delta 3}^{y},\;$ was excited at 4.12 GHz and 4.66 GHz, respectively. The CP response of the antenna is depicted in Figure 4, and, as shown, the 3-dB axial ratio bandwidth was nil as well. The results of this configuration were encouraging, but needed further modification and optimization to achieve the desired bandwidths for the targeted application. Geometry 2 was further modified, and the impact of the modification is discussed in the next section. 

### 3.3. Geometry 3

In geometry 3 the RDRA was energized by adding one more strip, as presented in Figure 2c. The modification is discussed in the next section. The return losses of this design configuration are depicted in Figure 3. The S_11_ curve of geometry 3 shows that the degenerate ${\mathrm{TE}}_{\delta 13}^{x}$ was excited at 4.16 GHz and ${\mathrm{TE}}_{1\delta 3}^{y}\;$ at 4.4 GHz, but still, impedance matching (|S_11_| ≤ 10 dB) was not achieved. As shown in Figure 4, the 3 dB axial ratio bandwidth of ~2% was achieved. The provided bandwidth was not enough to cover the targeted 5G band. Moreover, the S_11_ of the antenna needed to be optimized further. So, the shape of the feeding strip was further changed and this is discussed in the next geometry.

### 3.4. Geometry 4

The geometry 4 of RDRA is presented in Figure 2d. This design configuration was achieved by adding an additional length to the feeding strip. At this stage, the feeding strip was composed of 4 different lengths. The impedance matching (|S_11_| ≤ 10 dB) over a bandwidth of ~8.8% was attained by this configuration, as shown in Figure 3. The degenerate mode pair of the first higher-order, i.e., ${\mathrm{TE}}_{\delta 13}^{x}$ at 4.14 GHz and ${\mathrm{TE}}_{1\delta 3}^{y},$ at 4.55 GHz was excited to generate the circularly polarized response. The circular polarization over a bandwidth of ~5.8% was provided by the antenna. A significant improvement was observed in S_11_ and AR ratio bandwidths but, still, the achieved CP response was not enough to cover the desired bandwidth i.e., 4.4–4.8 GHz. This design configuration was again modified to make the final proposed geometry.

### 3.5. Geometry of the Proposed CP 5G RDRA

**Figure 5:**
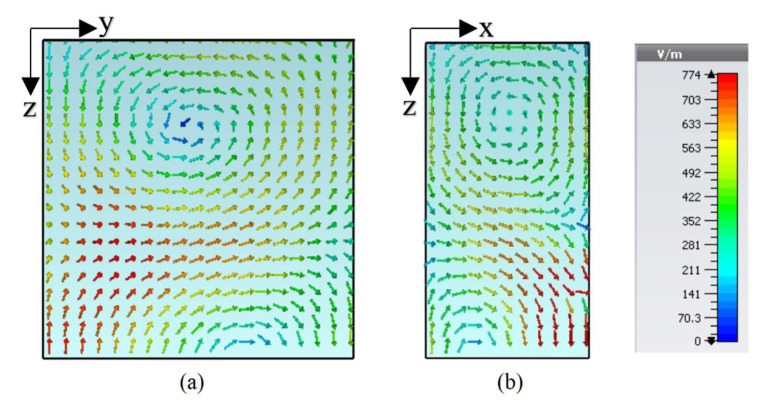
E-field distribution of the CP 5G RDRA (**a**) ${\mathrm{TE}}_{\delta 13}^{x}$ at 4.13 GHz; (**b**) ${\mathrm{TE}}_{1\delta 3}^{y}$ at 4.52 GHz.

**Figure 6:**
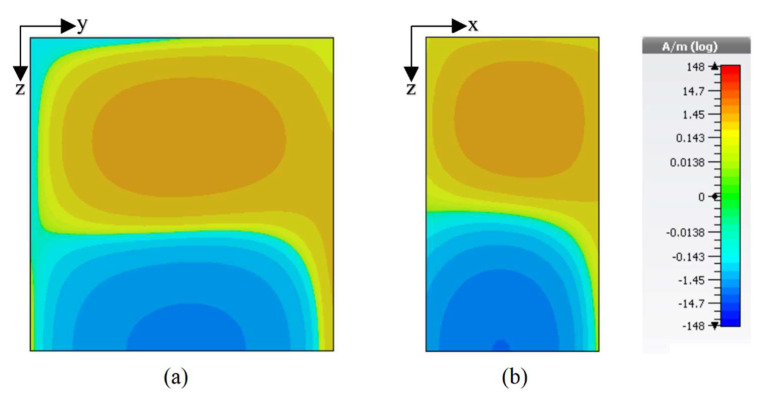
H-field distribution of the CP 5G RDRA (**a**) ${\mathrm{TE}}_{\delta 13}^{x}$ at 4.13 GHz; (**b**) ${\mathrm{TE}}_{1\delta 3}^{y}$ at 4.52 GHz.

The final design geometry was developed by adding the fifth and last length to the feeding strip to make the desired 5G Sub–6 GHz rectangular DRA, as depicted in Figure 1 and Figure 2b. The optimized strip lengths were $l_{1}=5.5$ mm, $l_{2}=7$ mm, $l_{3}=5$ mm, $l_{4}=10$ mm, and $l_{5}=1.5$ mm. The feed parameters were optimized by running a number of simulation sweeps to get the desired CP response. An impedance matching (|S_11_| ≤ 10 dB) over a bandwidth of ~17% was provided by the proposed geometry, as presented in Figure 3. As shown, the degenerate modes ${\mathrm{TE}}_{\delta 13}^{x}$ at 4.13 GHz and ${\mathrm{TE}}_{1\delta 3}^{y}\;$ at 4.52 GHz were excited to generate the circularly polarized response. The E-field and H-field distribution of the proposed CP 5G RDRA aare depicted in Figure 5 and Figure 6, respectively.

**Figure 7:**
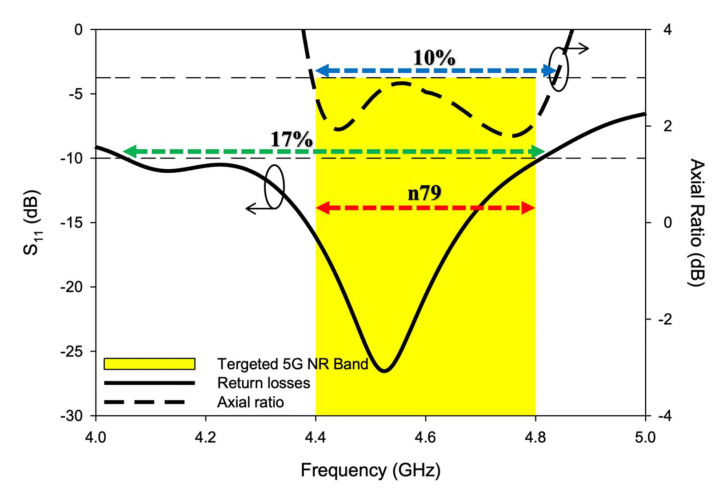
S11 and AR ratio overlapping bandwidths of the CP 5G RDRA.

**Figure 8:**
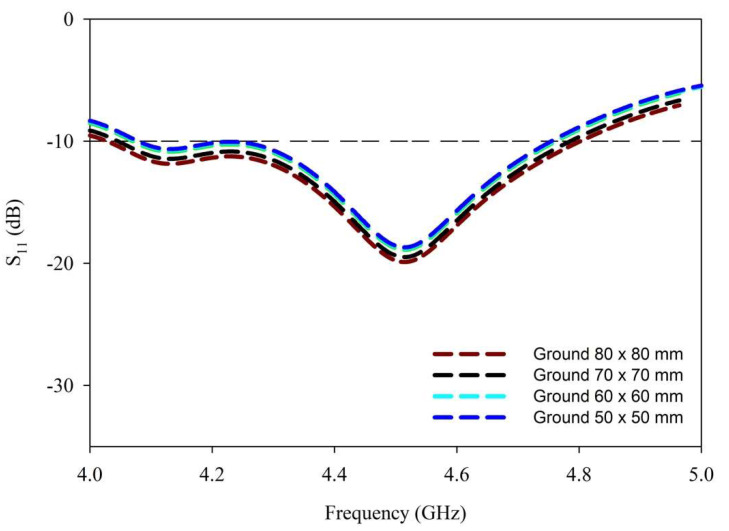
S11 of the CP 5G RDRA with different ground plane sizes.

**Figure 9:**
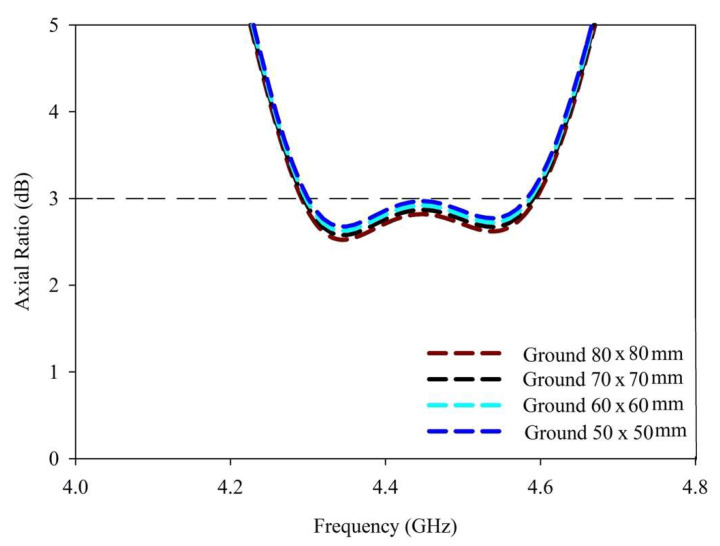
AR ratio of the CP 5G RDR with different ground plane sizes.

**Figure 10:**
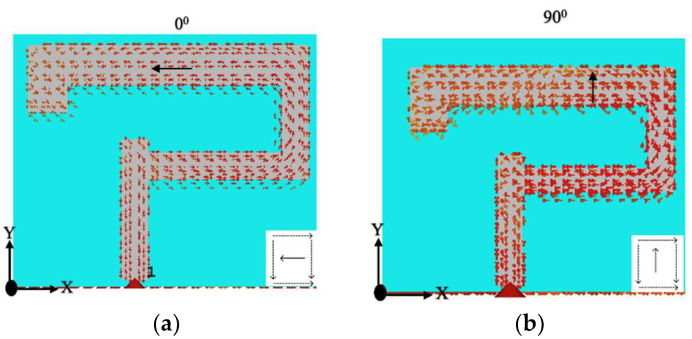
The surface current distribution at the novel feed of the CP 5G RDR. (**a**) At 0^o^; (**b**) At 90^o^.

The CP response over a bandwidth of ~10% was provided by the antenna. The achieved 3 dB axial ratio extended from 4.4–4.84 GHz which was good enough to cover the targeted n79 band. The AR and S11 bandwidths were achieved over the same range, as shown in Figure 7. Moreover, as the DRA radiated away from the ground the size did affect the performance much [26]. The antenna was simulated on different ground plane sizes and the results of S11 and the axial ratio are presented in Figure 8 and Figure 9. The simulated surface current distributions of the antenna at 4.13 GHz (Minimum of S11) are depicted in Figure 10. As can be seen the composite current surface currents on the novel feed were orthogonal at 0° and 90°, which provided the required condition for CP generation.

The wideband CP response and higher-order mode excitation were achieved using a low-cost simple design configuration without any complexity, which is a good contribution to those reported in the literature. The performance comparison of different geometries in the development of the desired CP 5G Sub–6 GHz antenna is summarized in Table 2. The optimized circularly polarized antenna was then fabricated to experimentally validate the proposed design which is demonstrated and explained in the next section.

## 4. Measurement Results

**Figure 11:**
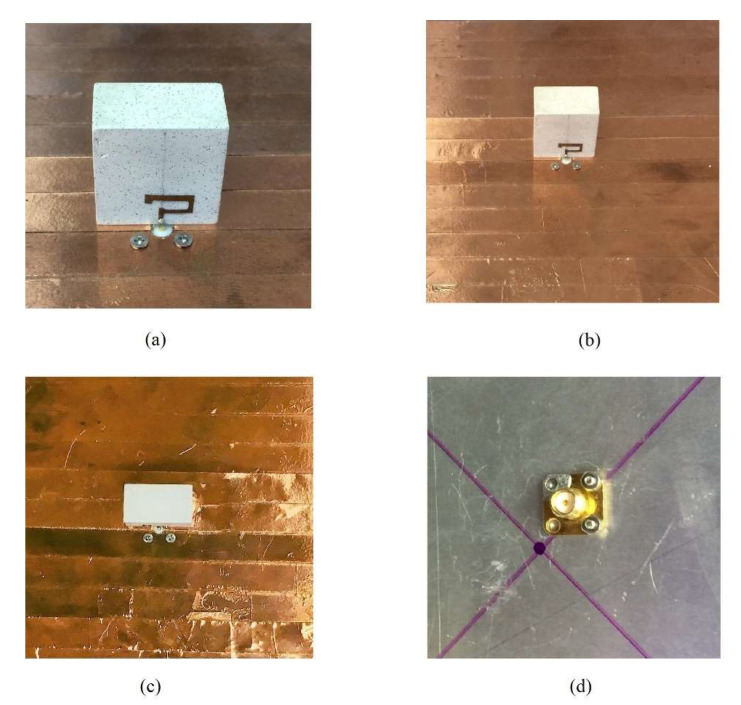
The geometry of the Proposed CP 5G RDRA Photograph of the prototype of the CP 5G RDRA. (**a**) Closeup; (**b**) Front View; (**c**) Top view; (**d**) Back view.

The optimized 5G NR band circularly polarized antenna was finally fabricated to measure the experimental results. The photographs of the proposed prototype are presented in Figure 11. The closeup, front view, top view, and back view of the antenna are depicted in Figure 11a–d, respectively. The ECCOSTOCK HiK with permittivity, $\varepsilon_{r}=10$ having loss tangent (δ) of 0.002 was used as DR material. An 80 × 80 mm aluminum ground plane was used. The feeding strip was cut from adhesive copper tape to easily stick to the surface of the DRA. An SMA was soldered at the feed point at the center of the DRA with the feeding strip. S_11_ was measured using a vector network analyzer (VNA), while far-field parameters were measured in the anechoic chamber. A 50-Ω coaxial cable was used to connect the SMA with the VNA. Double-sided copper tape was used to stick the DRA on the aluminum ground plane] to remove the possible air gap, according to the procedure explained in [26].

**Figure 12:**
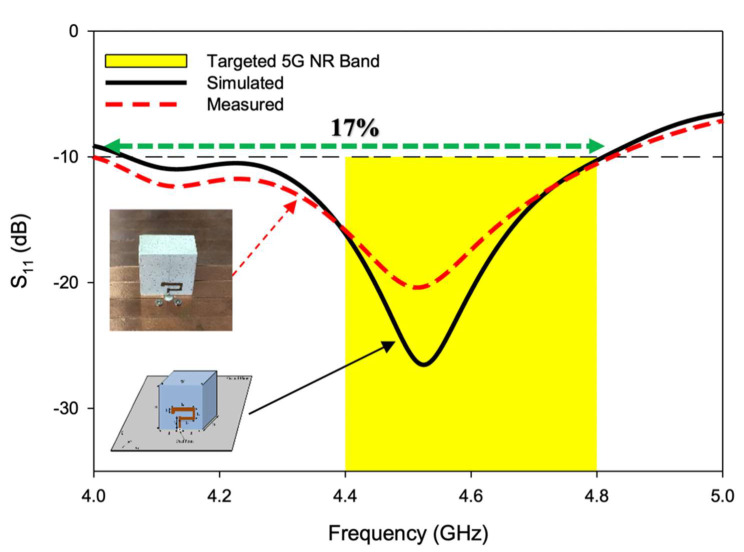
Simulated and measured S11 of the CP 5G RDRA.

The resonant mode frequencies of the RDRA could be predicted using mathematical equations of the dielectric waveguide model, as explained in [27]. According to DWM ${\mathrm{TE}}_{\delta 13}^{x}$ was estimated at 3.89 GHz and ${\mathrm{TE}}_{1\delta 1}^{y}$ at 4.53 GHz. The comparison between simulated and measured S_11_ of the CP DRA is presented in Figure 12.${\;\mathrm{TE}}_{\delta 13}^{x}$ was simulated at 4.13 GHz and measured at 4.12 GHz while ${\mathrm{TE}}_{1\delta 3}^{y}$ was simulated at 4.52 GHz and measured at 4.51 GHz. A close comparison was observed between predicted, simulated, and measured values. The comparison of these values is presented ed in Table 3. The impedance matching bandwidth (|S_11_| ≤ 10 dB) expanded from 4.05–4.81 GHz in simulation and 4.01–4.83 GHz in measurement. The antenna provided a measured S_11_ over a bandwidth of ~17%. A small marginal difference between simulation and measurement was observed, due to cable losses and measurement errors.

**Figure 13:**
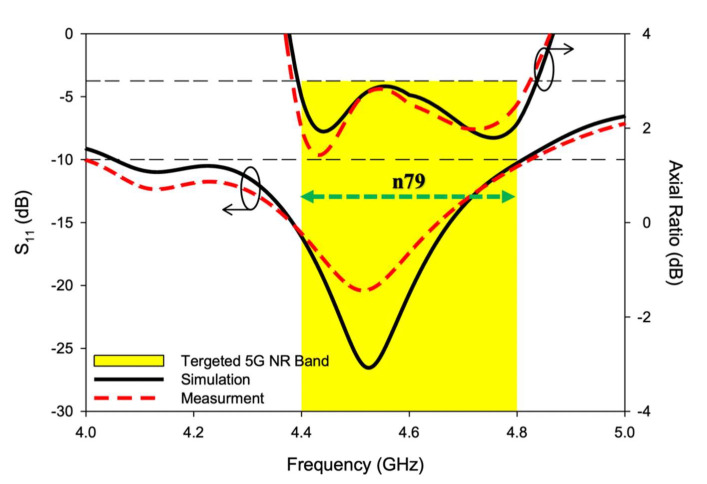
Simulated and measured axial ratio of the CP 5G RDRA.

**Figure 14:**
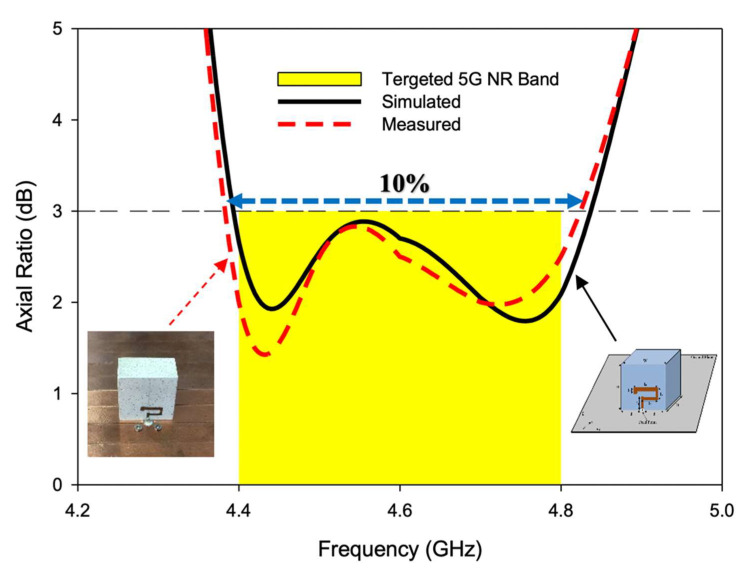
Simulated and measured S_11_ and AR overlapping bandwidths of the CP 5G RDRA.

Figure 13 shows the simulated and measured CP response of the proposed RDRA in the boresight direction (i.e., $Ɵ=0^{{}^{\circ}}$,$\;\Phi =0^{{}^{\circ}}$) As presented, the axial ratio extended from 4.4–4.84 GHz in simulation, while 4.38–4.82 GHz was measured during the experiment. Circular polarization of bandwidth of ~10% was achieved, both in simulation and measurement. The minimum AR value was simulated at 4.44 GHz and measured at 4.43 GHz with a magnitude of 1.92 dB and 1.4 dB, respectively. The negligible difference between theory and experiment was due to cable losses and measurement imperfections. The successful overlap of S11 and AR bandwidths is depicted in Figure 14. As shown, the overlapped bandwidth was good enough to cover the targeted 5G NR Sub-6 GHz band i.e., n79 (4.4–4.8 GHz). A stable and satisfactory performance was offered by the antenna during the experiment.

**Figure 15:**
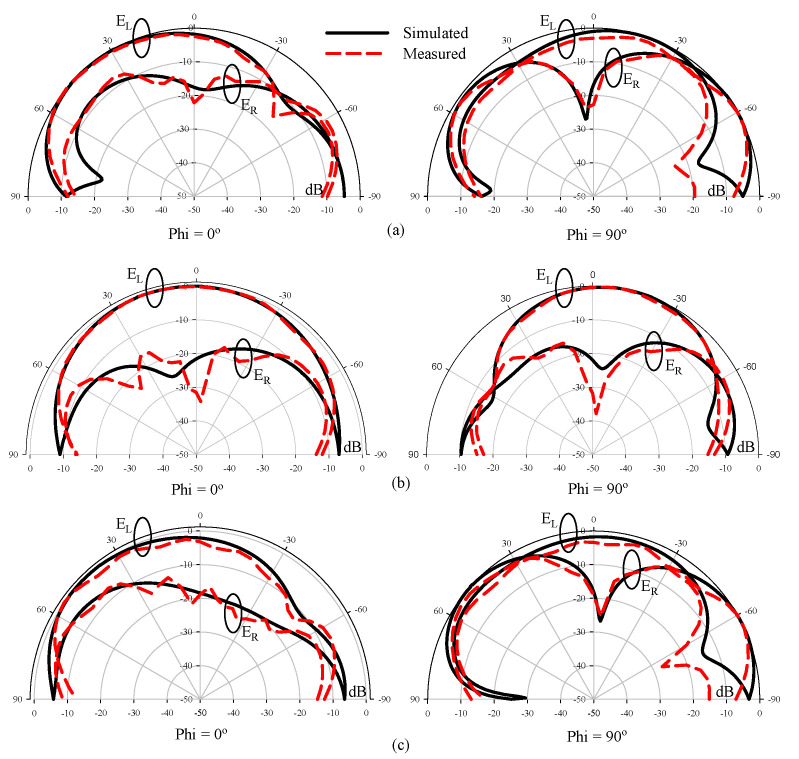
Simulated and measured radiation patterns of the CP 5G RDRA. (**a**) 4.4 GHz; (**b**) 4.43 GHz; (**c**) 4.7 GHz.

**Figure 16:**
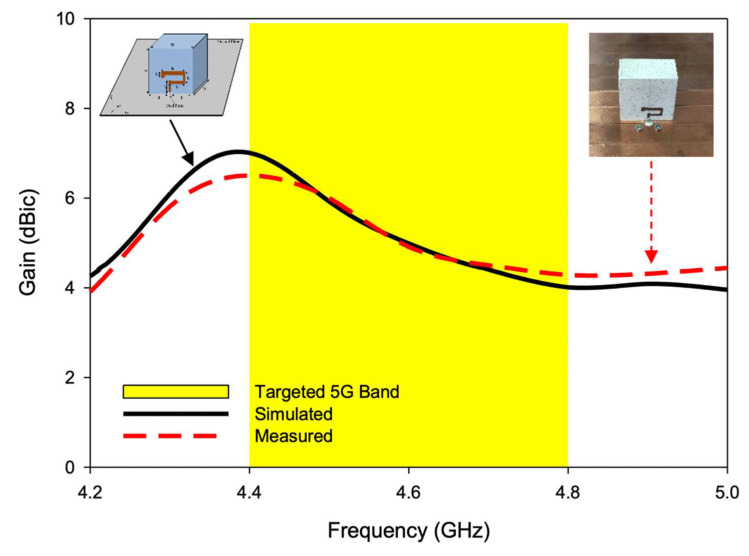
Simulated and measured gain of the CP 5G RDRA.

The simulated and measured radiation patterns of the proposed CP 5G RDRA are presented in Figure 15. The radiation patterns were computed and measured at three different frequencies. The antenna provided stable radiation patterns with left-hand circular polarization, since the left-hand field component was greater than the right-hand field component by a margin of more than 20 dB at minimum AR frequency i.e., 4.43 GHz, as shown in Figure 15b. The right-hand CP response could be achieved by reversing the feeding strip. The simulated and measured gain of the CP antenna are shown in Figure 16. The antenna offers a useful gain of ~6.2 dBic throughout the CP bandwidth. This high gain was achieved by excitation of the higher-order mode. A reasonable resemblance was observed between simulated and measured results.

In Table 4, the proposed CP 5G RDRA is compared with recently reported RDRAs in the literature. It can be concluded that the proposed design configuration offers wide CP bandwidth achieved by excitation of the orthogonal higher-order mode pair, with very simple design geometry. Basic DR shape, i.e., rectangular, was used as radiating element along with the implementation of a simple feeding mechanism. Moreover, the field distribution inside the DRA was controlled by short adjacent magnetic dipoles. The spacing between these dipoles was responsible for the gain of the DRA, which could be improved by increasing the spacing. The spacing could be enhanced by excitation of the DRA in higher-order mode [28]. In literature, different efforts have been made to excite the RDRA in higher-order mode for high gain applications but the reported geometries are complicated and not easy to implement. In the proposed antenna higher-order orthogonal mode was excited using a new conformal feeding strip that was cut from an adhesive copper tape, which is a simple and cost-effective solution to the problem.

## 5. Conclusions

This paper reported on a new low-cost circularly polarized RDRA for 5G NR Sub-6 GHz band applications. A new conformal metal strip was utilized for excitation of the higher-order orthogonal mode pair to generate CP response. A circular polarization over a bandwidth of ~10% was achieved along with a wide impedance matching bandwidth of ~17%. The axial ratio bandwidth was in conjunction with impedance matching bandwidth. A left-hand CP response was achieved with stable radiation patterns throughout the circular polarization bandwidth. A useful gain of ~6.2 dBic was attained by the antenna. The use of a simple and low-cost feeding mechanism, i.e., a unique conformal strip, to generate a circularly polarized wave by excitation of higher-order orthogonal modes, is the significant feature of this research, as compared to reports in the literature. A good agreement was observed in simulation and experimental results.

## Figures and Tables

**Table 1:** AR and S_11_ bandwidths for different feed dimensions of the proposed CP 5G RDRA.

*l_1_* (mm)	*l_2_* (mm)	*l_3_* (mm)	*l_4_* (mm)	*l_5_* (mm)	3 dB AR Bandwidth (%)	10 dB S11 Bandwidth (%)	Overlapping AR & S11 Bandwidth (%)
3.5	7	1	8	1	6.24	12.43	100
		2	8	0.5	5.65	12.18	100
	9	1	12	0.5	6.23	11.92	100
				1	6.64	10.65	100
				1	7.87	10.4	100
		2	12	2.5	6.17	10.14	93.1
				3	7.08	11.67	76.2
				1.5	9.07	11.42	66.2
		3	10	2.5	5.64	11.16	90.5
				3	6.28	8.63	86.3
				2	7.25	8.38	100
**5.5**	**7**	3	7	1.5	4.17	9.89	100
				1	5.32	9.63	100
				2.5	7.59	9.38	100
		4	8	3.5	7.43	9.13	100
		**5**	**10**	**1.5**	**10**	**17**	**100**
	9	5	12	2	5.07	7.9	48.3
7.5	5	5	8	4	6.15	7.42	77.5
		6	6	3.5	4.71	7.2	100
				2.5	7.08	6.97	43.4
		7	9	2	6.15	6.75	100
				2	6.1	6.54	100
	7	7	8	1.5	5.3	6.34	100
				3.5	10.47	10.91	60.3
	9	6	10	3	4.05	7.66	100

**Table 2:** Performance comparison of different geometries in the development of the CP 5G RDRA.

Antenna	Geometry	No of Strips	10-dB S_11_ Bandwidth (%)	3-dB AR Bandwidth (%)	Polarization	5G NR n79 Coverage (4.4–4.8 GHz)
Geometry 1	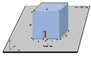	1	Nil	Nil	Linear	NO
Geometry 2	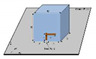	2	Nil	Nil	Circular	NO
Geometry 3	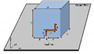	3	Nil	2	Circular	NO
Geometry 4	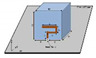	4	8.8	5.8	Circular	NO
Proposed CP 5G RDRA	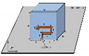	**5**	**17**	**10**	**Circular**	**YES**

**Table 3:** Comparison between predicted, simulated, and measured mode frequencies of the CP 5G RDRA.

Excited Mode of 5G CP RDRA	DWM Estimation	CST Calculation	Experimental Measurement
*TE*	$f_{DWM}$ (GHz)	$f_{CST}$ (GHz)	$f_{Measurment}$ (GHz)
${\mathrm{TE}}_{\delta 13}^{x}$	3.89	4.13	4.12
${\mathrm{TE}}_{1\delta 3}^{y}$	4.53	4.52	4.51

**Table 4:** Comparison between proposed CP 5G RDRA with recently reported RDRAs in literature.

Ref.	$\varepsilon_{r}$	Design Configuration	Excitation Mechanism	Mode of Excitation	Usable CP Frequency	Usable CP Bandwidth	Gain (dBic)	Antenna Geometry
[18]	10 & 3.5	Rectangular multilayered DRA	Cross-slot with microstrip line	${\mathrm{TE}}_{111}$ & ${\mathrm{TE}}_{113}$	10.5−11.5 GHz	9.5%	11	Complex
[19]	10	Four metallic walls surrounded RDRA	Cross-slot with microstrip line	${\mathrm{TE}}_{111}$	2.2−3.5 GHz	46.9%	4.37	Complex
[20]	9.6	Rectangular DRA with two annular vias	Microstrip line coupled rectangular slot	${\mathrm{TE}}_{113}$	3.28−3.46 GHz	5.5%	6–7.1	Complex
[21]	9.8	Rectangular DRA	Triangular aperture with parasitic strip	${\mathrm{TE}}_{111}$	3.46−3.54 GHz	2.29%	5	Complex
[22]	20.5	Rectangular DRA	Cross-slot-coupled	${\mathrm{TE}}_{111}$	1.25−1.3 GHz	2.2%	4.3	Complex
Proposed CP 5G RDRA	**10**	**Rectangular DRA**	**Unique conformal strip**	** $TE_{113}$ **	**4.4** **−** **4.8 GHz**	**10%**	**6.2**	**Simple**

## Data Availability

All data has been included in the study.
